# New Zealand’s emergency department target – did it reduce ED length of stay, and if so, how and when?

**DOI:** 10.1186/s12913-017-2617-1

**Published:** 2017-09-26

**Authors:** Tim Tenbensel, Linda Chalmers, Peter Jones, Sarah Appleton-Dyer, Lisa Walton, Shanthi Ameratunga

**Affiliations:** 1 Health Systems, School of Population Health, Faculty of Medical and Health Sciences, University of Auckland, Private Bag, 92019, Auckland, 1142 New Zealand; 2 Nursing Development Unit, Auckland City Hospital, Private Bag 92024, Auckland, 1142 New Zealand; 3 Adult Emergency Department, Auckland City Hospital, Private Bag 92024, Auckland, 1142 New Zealand; 4 Epidemiology and Biostatistics, School of Population Health, Faculty of Medical and Health Sciences, University of Auckland, Private Bag, 92019, Auckland, 1142 New Zealand

**Keywords:** Hospital emergency departments, Crowding, New Zealand, Targets, Short-stay units, Patient flow, Mixed methods

## Abstract

**Background:**

In 2009, the New Zealand government introduced a hospital emergency department (ED) target – 95% of patients seen, treated or discharged within 6 h - in order to alleviate crowding in public hospital EDs. While these targets were largely met by 2012, research suggests that such targets can be met without corresponding overall reductions in ED length-of-stay (LOS). Our research explores whether the NZ ED time target actually reduced ED LOS, and if so, how and when.

**Methods:**

We adopted a mixed-methods approach with integration of data sources. After selecting four hospitals as case study sites, we collected all ED utilisation data for the period 2006 to 2012. ED LOS data was derived in two forms-*reported* ED LOS, and *total* ED LOS - which included time spent in short-stay units. This data was used to identify changes in the length of ED stay, and describe the timing of these changes to these indicators. Sixty-eight semi-structured interviews and two surveys of hospital clinicians and managers were conducted between 2011 and 2013. This data was then explored to identify factors that could account for ED LOS changes and their timing.

**Results:**

Reported ED LOS reduced in all sites after the introduction of the target, and continued to reduce in 2011 and 2012. However, total ED LOS only decreased from 2008 to 2010, and did not reduce further in any hospital. Increased use of short-stay units largely accounted for these differences. Interview and survey data showed changes to improve patient flow were introduced in the early implementation period, whereas increased ED resources, better information systems to monitor target performance, and leadership and social marketing strategies mainly took throughout 2011 and 2012 when total ED LOS was not reducing.

**Conclusions:**

While the ED target clearly stimulated improvements in patient flow, our analysis also questions the value of ED targets as a long term approach. Increased use of short-stay units suggests that the target became less effective in ‘standing for’ improved timeliness of hospital care in response to increasing acute demand. As such, the overall challenges in managing demand for acute and urgent care in New Zealand hospitals remain.

**Electronic supplementary material:**

The online version of this article (10.1186/s12913-017-2617-1) contains supplementary material, which is available to authorized users.

## Background

Overcrowding and delays to treatment for patients in emergency departments (EDs) are health system problems associated with poor patient, ED and hospital service outcomes across the world [1–7]. In New Zealand, problems with the quality (including timeliness) of ED services emerged in the mid-1990s. In 2007–2008, ED crowding became increasingly apparent in New Zealand as a problem requiring attention [8].

New Zealand’s hospital services are predominantly funded and provided publicly, apart from a small private hospital sector that specialises in elective surgical procedures. Public hospital services are controlled by, and accountable to central government through local health provider organisations known as District Health Boards (DHBs) [9]. In July 2009, the New Zealand government introduced the ‘Shorter Stays in ED’ target which required that in all DHBs, 95% of patients would be admitted, discharged, or transferred from an ED within 6 h [10].

ED targets remain a controversial policy instrument, and debates persist about the potential for positive and negative consequences beyond their intended effects [11–14]. As a persistent question about ED targets is whether they are actually successful in improving timeliness of care and treatment [12, 15], in this paper, we focus on whether or not targets stimulated reductions in ED length of stay. This paper is part of a wider research project investigating New Zealand’s ED time target, and research protocols for this project are outlined in another paper [16]. Other important questions about the implementation of the NZ ED target that our research team has already, or is planning to address elsewhere include the effects of the target on ED quality [16–19], the effects on the division of labour within hospitals [20] and the costs of implementing the target [21].

### ED crowding – Causes and possible solutions

Research into ED crowding has highlighted a range of contributing factors. Increasing demand for ED services often exceeds population increases [22]. Drivers of this increasing demand for emergency care include growing and ageing populations, increasing incidence of long term conditions, gaps in primary care service delivery, and challenges in aged residential care management [1, 8]. In addition to these utilisation trends, the ways in which ED and wider hospital services are configured can also contribute to ED crowding. Lack of available hospital inpatient beds for acute admissions from ED is the principal cause of access block that in turn results in ED crowding and prolonged stays for patients in the ED [3]. In New Zealand, the Ministry of Health identified additional issues regarding ED length of stay including problems with triage processes, insufficient ED beds and inadequate ED staffing [8].

Initiatives to tackle ED LOS and crowding can originate from within EDs, from central government, and anywhere in-between. Some researchers [23, 24] distinguish between ‘input’, ‘throughput’ and ‘output’ solutions. Input solutions are aimed at managing increasing ED demand by providing alternative care paths such as more accessible primary care on evenings and weekends. Throughput solutions ‘focus on managing staff, space and processes within the ED better’ [24]. ‘Output’ solutions focus on improving flow to inpatient wards or discharge. More broadly, organisational approaches available to hospital and healthcare management include changes to inpatient wards processes and flows. Organisations can also adopt information technologies to track patient flow and bottlenecks [1, 25], and can seek to invoke leadership and/or cultural change as a way of promoting changes in practices within hospitals [26].

In health systems in which hospitals are publicly funded and operated, the use of time targets as an instrument for reducing ED crowding and length of stay has become common since 2000. The Accident & Emergency (A&E) target introduced in England in 2001 required hospitals to see, treat or discharge 98% of patients within four hours [27, 28]. This policy approach, has since spread to New Zealand [29, 30], Australia [31, 32] and Canada [33] albeit with different parameters around time and required levels of performance. The rationale behind targets is that health organisations will be incentivised to develop their own solutions and initiatives to meet the target, and therefore alleviate problems of ED crowding and timeliness of treatment.

### Do time targets reduce ED length of stay?

A considerable body of research on the use of time targets in Emergency Departments has developed since the early 2000s. Central to this literature is the experience of the English A&E target in the early 2000s. English health care organisations responsible for hospitals faced sanctions for poor performance in terms of this and a range of other targets and performance indicators. Many commentators on the English case have argued that this target resulted in considerable improvements in quality and timeliness of emergency department treatment [34–36]. These authors compare the dramatic improvements in timeliness in England with the lack of improvement in Scotland and Wales where the target did not apply.

Research into Australia’s National Emergency Access (four hour) Target (NEAT) also shows some improvements attributable to the introduction of time targets in 2009 [37, 38]. However, a key difference between the English and Australian targets is the degree of pressure placed on hospitals. In Australia, the effects of NEAT varied considerably between states, reflecting different degrees of emphases from state governments [39].

New Zealand’s official target performance data shows that the target level of performance was achieved, or very nearly achieved by every DHB by 2012 [14, 40]. While there were no formal sanctions for poor performance, the Minister of Health and the Ministry placed considerable pressure on the DHBs to achieve the ED target as part of a wider regime of target-based accountability [14]. These measures were primarily informal, such as phone calls to DHB chief executives when target performance was deemed below par.

From a research perspective, however, relying only on official target performance data to provide evidence of improved timeliness is problematic. The first problem is that a performance measure may not adequately ‘stand for’ the desired objective [11, 41]. The time-to-complete-treatment target, expressed as a percentage seen within a certain period, does not capture the range of quality dimensions of ED services, such that it is possible that improvements in timeliness could be achieved at the expense of other aspects of clinical quality [42].

Secondly, in situations in which those responsible for implementing the target are also in charge of the data collection, there is the possibility that such data may be partially fabricated or massaged [43]. Audits of the English A&E target produced very different figures of target performance than the data collected by hospitals themselves [13, 44]. The data control problem is exacerbated in environments in which the achievement of the target is ‘high-stakes’ for organisations held publicly accountable for achievement, and explicitly linked to the electoral ambitions of governing political parties [14]. This is a pervasive feature of performance management in the public sector [41].

If it is not appropriate to use the target measure itself as a proxy for successful reductions in ED length of stay (LOS), it is necessary to find alternative metrics for reduced ED LOS. Mason et al. [2] in their analysis of the English A&E target data use median ED LOS as their proxy for timeliness [2]. In their study of 15 English A&Es from 2003 to 2006, there were reductions in median ED LOS from 2003 to 2004, but not after 2004, even though target performance improved after 2004 [2]. This alternative metric is not necessarily superior to official target measures as it too will have limitations – as with the time target, it gives no information about quality. Nevertheless, the median ED LOS provides a simple and robust means of checking claims about timeliness. Mason et al. [2] showed that English A&E patients admitted to wards experienced progressively *longer* median waiting times in A&E each year, and concluded on this basis that the English target was not a reliable indicator of timeliness.

Finally, the value of official ED target performance figures may also be problematic in the context of growing use of short-stay units (SSUs) within EDs or in other parts of the hospital. These units, (acute assessment units, observation wards), have been increasingly established and used by hospitals as part of a strategy to manage acute demand [45, 46]. The increasing role of SSUs can be justified on clinical and organisational grounds [47, 48]. Nevertheless, the existence of these units has the potential to confound ED LOS data, particularly if patients referred to SSUs are taken ‘off the target stream’ [31].

## Methods

Our central research questions are:

1. Did Emergency Department length of stay reduce over the 2009–2012 period as a consequence of the target, and if so, when?
2. Which actions taken by hospitals can account for ED LOS reductions (if apparent)?

### Overall approach

Our overall approach involved a mixed methods design that integrated case study data from four hospital sites. Data sources included data on ED length of stay pre and post target implementation, and qualitative data from interviews and surveys of key informants (managers and clinicians) in each case study setting. The methods remained distinct throughout the data collection phase, with mixing occurring at the level of interpretation and developing conclusions [49]. Analysing ED length of stay alongside information on the actions taken at each of the hospitals supported us in making attributions relating to ED length of stay and specific actions and/or types of actions taken at the hospitals.

### Case study design

We adopted a multiple, comparative case study approach in order to distinguish between common and context-specific factors. Four case study sites were selected to ensure variation in demography, hospital size, and number of ED presentations. Larger, urban and regional hospitals are over-represented in our case studies because these are the hospitals which were more likely to manifest the problems of crowding that prompted the introduction of the target [20, 29]. In addition two of the case study hospitals had poorer performance on the target measure at the time the target was introduced to the sector (below 80% at first quarter reporting in 2009). Collectively, our four case study hospitals had catchment areas covering over 25% of the New Zealand population, and 26.5% of total ED presentations in New Zealand over the 2006–2012 period. As shown in Table 1 below, our case study sites vary in the background patterns of demand. For one hospital, increases in ED presentation were relatively low after 2010, which is most likely attributable to a lower rate of population growth. Each of the three other case sites experienced increases in demand of between 3 and 8% each year. The four hospital sites are described individually in the results section.

**Table 1:** ED presentations and increases in case study hospitals, 2009–2012

	District population size (2013)	% District population growth 2006–13^a^	No. of ED presentations and annual percentage increase 2009–2012						
			2009	2010	% Increase 2009–10	2011	% Increase 2010–11	2012	% Increase 2011–12
Hospital 1	100–200,000	2.32%	35,608	37,503	5.32%	38,221	1.91%	38,556	0.88%
Hospital 2	>400,000	8.36%	87,706	93,804	6.95%	98,103	4.58%	101,457	3.42%
Hospital 3	200–400,000	5.93%	54,233	58,157	7.24%	62,933	8.21%	66,680	5.95%
Hospital 4	>400,000	9.12%	52,645	56,159	6.67%	59,756	6.41%	62,120	3.96%
Total	N/A	7.40%	230,192	245,623	6.70%^2^	259,013	5.45%^2^	268,813	3.78%^2^

^a^Statistics New Zealand: http://m.stats.govt.nz/Census/2013-census/data-tables/dhb-tables.aspx

^b^Weighted % increase across the four hospital sites

### Data sources

#### ED length of stay

To determine ED LOS, all ED visits and hospital admissions (collectively termed ‘events’) from 1/1/2006 to 31/12/2012 were identified from the central database of the New Zealand Health Information Service (NZHIS). The visit date, demographic data and date of death were extracted from NZHIS and then linked to the individual case site databases holding times for the patient journey (presentation, triage, assessment, admission and discharge times) for each event using a unique patient identifier, the National Health Index (NHI) number. Duplicate events were identified and removed prior to data analysis. The ED LOS was calculated as the interval between ED presentation time and ED departure time. Two ED LOS variables were calculated. Firstly we use the ***reported*** ED LOS, which does not include the time spent in an ED short-stay unit (SSU). This is what is reported to the Ministry of Health by DHBs to check compliance with the target and is the basis for our report of target performance at the case sites. We note that this is not the same as publicly reported data for DHBs, because many DHBs have more than one hospital.

Secondly we use the ***total*** ED LOS, which includes, includes the time spent in a SSU as ED time. Although it was intended that the SSU for the purposes of this study would be an ED SSU, not all sites were able to provide data in a format that separated ED SSU from acute medical or surgical SSU. This data was collected from all New Zealand hospitals, but only the data pertaining to the four case study hospitals is drawn on in this analysis. We follow Mason and colleagues [50, 51] in using the median with interquartile range (IQR) for reported and total ED LOS, as this provides an alternative indicator of timeliness of treatment in EDs. The median (IQR) is more appropriate than the average because the distribution of ED LOS is right-skewed. As such, the median figure is highly unlikely to be affected by any distortions to ED LOS data attributable to staff avoiding target breaches at the 6 h point.

We have used descriptive statistics to portray trends in ED length of stay over the 2007 to 2012 period as these are sufficient to depict changes. For this data, tests of statistical significance of changes will not add value to the picture because with the large numbers involved, any year-on-year difference is likely to be statistically significant at the *p* < 0.001 level.

#### Case study site interview and ED survey

Sixty eight face to face semi-structured interviews were conducted with clinical and management staff in the ED and wider hospital over two rounds of data collection in February to July 2011 (47 interviews) and April to July 2012 (21 interviews), with thirteen participants interviewed in both rounds (see Additional file 1 for the interview schedule). Thirty-six of the interviews were with clinicians (nursing, medical and allied health), 10 with clinical managers, and 22 with non-clinical mangers in the hospital and DHB organisation. Twenty-nine interviews were with staff located within EDs, and 39 with staff in the hospital and wider DHB. A small number of documents were also collected from each of the study hospitals [20]. In addition, surveys of the Clinical Directors and Service Managers of all EDs in New Zealand, which included open-ended questions, were undertaken in 2011 and 2013 to determine the interventions and resources used to help meet the target (the full survey methodology and survey tool have been published previously) [21]. The data from these surveys pertaining to the four case study sites these surveys was used to inform the current paper see Additional file 2 for details of the survey questions.

From the interviews and the survey we compiled a spreadsheet that identified the strategies and mechanisms adopted at each case study site. Information in this spreadsheet formed the basis of Table 5 shown in our Results section. The categorisation of activities used for this table was developed inductively and iteratively by one author (LC) who conducted all the interviews and analysed this data alongside the information from the surveys.

### Integration of sources

The research questions were used as key themes to guide the integration of the data sources within and across the four hospital sites. The ED LOS data was used to identify whether there were reductions in the length of ED stay, and describe the timing of any changes to these indicators. We then explored data from the DHB surveys and interviews to identify common and site-specific factors that could account for these changes and their timing. To support this, an integrated data display was used to display the findings from each of the data sources. This allowed us to iteratively develop a small number of broad categories of target-related initiatives that could be used to classify data from the surveys and interviews across all cases. Similar approaches have been used in other mixed methods studies [52, 53]. This also enabled the interview and survey data to thematically analysed to identify key categories of actions undertaken at the hospitals.

## Results

### The four hospital sites and their official target performance

Hospital 1 (H1) is a midsized provincial hospital in a district with relatively low rates of population growth (see Table 1 above). In the quarter prior to target implementation (April to June 2009), about 80% of ED patients were seen, treated or discharged within 6 h. Target performance reached 90% in January to March 2010, and 95% in October to December 2011.

Hospital 2 is a large urban hospital that from the outset had advanced quality improvement capability and experience. Reduction of ED length of stay had been identified as an organisational priority two years prior to the introduction of the national target. Its ‘pre-target’ performance was 80% and it quickly achieved 90% in the first quarter of the implementation period (July to September 2009), and first met the target of 95% in early 2010.

Hospital 3 is a large hospital based in a major regional centre. This site has the additional complexity of acute service responsibilities for other hospitals in the DHB and the provision of a range of specialised clinical services to other DHBs. A new ED and SSU had been planned for this site prior to the target, and these were to open in 2011 and 2012. At this site, pre-target performance was below 65%, and by the end of the research period, its official target performance was comparatively low at around 85%, without ever having reached the 95% target.

**Fig. 1:**
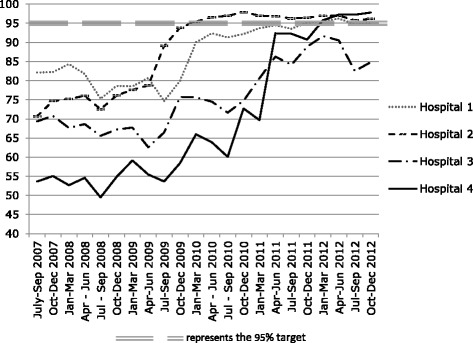
(Case study hospital target performance 2007–2012)

Hospital 4 is a large urban hospital, where a new ED and associated SSU had also been planned prior to the target, and were due to open in 2011. Its performance on the target measure prior to implementation was very low (55%), rising to 72% in January to March 2011, then rapidly to 92% the following quarter, finally achieving the target in early 2012. Figure 1 below shows the target performance of each case study hospital from mid-2007 (two years prior to target implementation) to the end of 2012. It is important to note that this data includes information from individual hospitals rather than DHBs (some of which have more than one hospital).

**Fig. 2:**
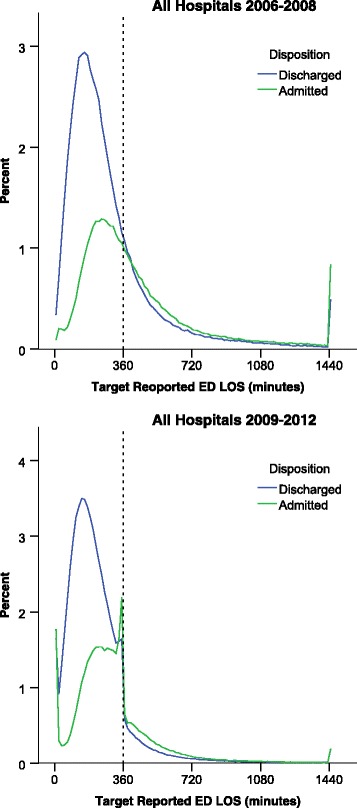
(Distribution of reported ED LOS before (2006–2008) and after (2009–2012) target implementation)

Figure 2 below shows the distribution of reported ED LOS times before and after the introduction of the target. The change in distributions indicates a significant change between the two time intervals. This figure distinguishes between patients discharged from ED, and those admitted to inpatient wards. The changes for the latter category are more marked. It is also apparent from this figure that a far larger proportion of discharged ED patients are treated within six hours (before and after target implementation), and that patients admitted to wards are more likely to wait more than six hours. For ease of presentation, our data in the remaining part of this section reports on the whole ED population, instead of distinguishing between discharged and admitted.

### Did ED LOS reduce, and if so, when?

#### Trends in reported and Total ED LOS

Leaving official target performance data to one side for the reasons outlined above, we need to establish whether ED LOS really did improve, and if so, to what extent. Following Mason et al. [2], we use median reported ED LOS to determine whether there were improvements in timeliness, with figures for the 25th and 75th percentile provided to give an indication of the spread. In Table 2 below, H1 and H2 show decreasing reported median ED LOS prior to 2010, with the reduction commencing in H2 well before the implementation of the target. In contrast, the largest reductions in H3 and H4 – both with higher median reported ED LOS prior to the target - occur after 2010.

**Table 2:** Quartiles of reported ED length of stay 2007–2012

Case study Hospital	Quartiles of **Reported** ED length of stay (hours)					
Year	2007	2008	2009	2010	2011	2012
Hospital 1	25th: 2.0 **Med: 3.5** 75th: 5.4	2.0 **3.4** 5.5	2.0 **3.6** 5.8	1.8 **3.0** 4.6	1.8 **3.1** 4.6	1.9 **3.0** 4.5
Hospital 2	25th: 2.7 **Med: 4.2** 75th: 6.4	2.6 **4.0** 6.0	2.4 **3.7** 5.2	2.3 **3.4** 4.7	2.3 **3.5** 4.8	2.3 **3.4** 4.7
Hospital 3	25th: 3.0 **Med: 4.5** 75th: 6.7	2.9 **4.6** 6.9	2.9 **4.5** 6.8	2.7 **4.2** 6.1	2.4 **3.7** 5.3	2.2 **3.6** 5.2
Hospital 4	25th: 3.1 **Med: 5.3** 75th: 9.6	3.4 **5.7** 9.8	3.3 **5.4** 8.6	2.9 **4.7** 7.3	2.3 **3.7** 5.4	2.2 **3.6** 5.0
All case study hospitals	25th: 2.7 **Med: 4.3** 75th: 6.8	2.7 **4.3** 6.8	2.6 **4.1** 6.2	2.4 **3.7** 5.4	2.1 **3.4** 4.9	2.0 **3.3** 4.8

Table 2 also illustrates that the trends in *reported* ED LOS closely match the trends in target performance (Fig. 1), with the reductions in all case hospitals occurring from 2009 to 2010, matching the increases in target performance, and with H3 and H4 showing further reductions in reported ED LOS in 2011, matching their increased target performance during this time.

A different picture emerges for ***total*** median ED LOS. This figure, which includes ED short-stay units, also reduced across the three hospital sites (H2, H3 & H4) for which our data included some information about short-stay units. However, the total ED LOS reductions were smaller than those observed for the reported ED LOS. In Table 3 we see reductions in median total ED LOS across all sites from 2009 to 2010 (the early implementation period), with the trend having begun earlier in H2. Reductions in the 75th percentile figure during this period are even more marked in each case study hospital. However, in each case, there is no sustained reduction in median total ED LOS after 2010. In H3 and H4, the total ED LOS median increased from 2011 to 2012, and a similar ‘bounce’ is apparent in H2 from 2010 to 2011. The figures for the 75th percentile (the higher figure in the inter-quartile range) also do not reduce further after 2010, with H4’s figure reverting to the alarmingly high 2008 level of 12.9 h.

**Table 3:** Quartiles of total ED length of stay (2007–2012)

Case study Hospital	Quartiles of Total ED length of stay (hours)					
Year	2007	2008	2009	2010	2011	2012
Hospital 1	25th: 2.0 **Med: 3.5** 75th: 5.4	2.0 **3.4** 5.5	2.0 **3.6** 5.8	1.8 **3.0** 4.6	1.8 **3.1** 4.6	1.9 **3.0** 4.5
Hospital 2	25th: 2.9 **Med: 4.7** 75th: 8.2	2.8 **4.6** 8.1	2.8 **4.4** 7.7	2.7 **4.2** 6.8	2.8 **4.5** 7.7	2.8 **4.5** 7.5
Hospital 3	25th: 3.0 **Med: 4.5** 75th: 6.7	2.9 **4.6** 6.9	2.9 **4.5** 6.8	2.7 **4.2** 6.1	2.7 **4.1** 6.0	2.7 **4.2** 6.0
Hospital 4	25th: 3.3 **Med: 6.2** 75th: 13.9	3.6 **6.4** 12.9	3.6 **6.1** 11.1	3.2 **5.4** 9.8	2.8 **5.0** 9.9	2.9 **5.4** 12.9
All case study hospitals	25th: 2.8 **Med: 4.6** 75th: 8.0	2.8 **4.6** 8.0	2.8 **4.6** 7.7	2.6 **4.2** 6.6	2.6 **4.2** 6.7	2.6 **4.3** 6.9

The differences between reported and total ED LOS for each hospital are shown in Table 4. For H2, H3 and H4, there were increasing differences between reported and total median ED LOS figures after 2010 (the late implementation period), indicating increased use of SSUs. The extent of these differences between reported and total median ED LOS at the end of the period (2012) varies from 0.6 h in H3 to 1.9 h in H4, with H2 showing a difference of 1.0 h. These differences between case study hospitals are more pronounced for the 75th percentile (H3: 0.8 h; H2: 2.7 h; H4: 7.8 h). To summarise, reported ED LOS reduced in all sites after the introduction of the target, and continued to reduce over time, though less steeply after 2010. However, total ED LOS only decreases until 2010. The total median ED LOS figures were no lower in 2012 than they were in 2010 in any case study hospital.

**Table 4:** Difference between total ED length of stay and reported ED length of stay (2007–2012)

Case study Hospital	Difference between Total ED length of stay and Reported ED length of stay (hours)					
Year	2007	2008	2009	2010	2011	2012
Hospital 1	25th: 0.0 **Med: 0.0** 75th: 0.0	0.0 **0.0** 0.0	0.0 **0.0** 0.0	0.0 **0.0** 0.0	0.0 **0.0** 0.0	0.0 **0.0** 0.0
Hospital 2	25th: 0.2 **Med: 0.5** 75th: 1.8	0.3 **0.6** 2.1	0.4 **0.8** 2.5	0.4 **0.8** 2.1	0.5 **1.0** 2.8	0.5 **1.0** 2.7
Hospital 3	25th: 0.0 **Med: 0.0** 75th: 0.0	0.0 **0.0** 0.0	0.0 **0.0** 0.0	0.0 **0.0** 0.0	0.3 **0.4** 0.7	0.4 **0.6** 0.8
Hospital 4	25th: 0.3 **Med: 0.9** 75th: 4.3	0.2 **0.8** 3.1	0.3 **0.7** 2.5	0.3 **0.7** 2.5	0.6 **1.3** 4.5	0.7 **1.9** 7.8
All case study hospitals	25th: 0.1 **Med: 0.3** 75th: 1.1	0.1 **0.3** 1.1	0.2 **0.5** 1.5	0.2 **0.5** 1.2	0.5 **0.8** 1.8	0.7 **1.0** 2.2

#### Trends in the use of short-stay units

**Fig. 3:**
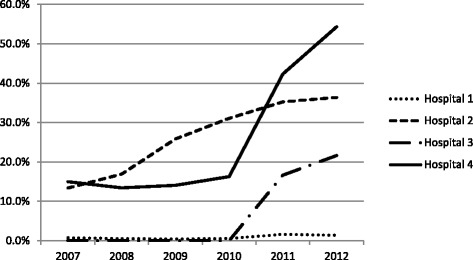
Reported and Total Median ED LOS in four case study hospitals

This information points to the crucial role played by short-stay units in enabling hospitals to meet the target. This is also confirmed by the data we have on short-stay utilisation, and their role in mediating the journey from ED inpatient wards. Figure 3 shows the proportion of ED patients eventually admitted to inpatient wards that did so via short-stay units. For H2, this percentage begins to increase before the introduction of the target. For H3 and H4, however, we see very large increases in this proportion after 2010.

These dramatic increases in the use of short-stay units in H3 and H4 correspond to the rapid increases in official target performance displayed in Fig. 1, (bearing in mind that target performance is recorded quarterly, whereas the data in Fig. 3 is annualised).

### Case study site interview and ED survey data: What did hospitals do to reduce ED length of stay?

The data reported above show a clear pattern in the timing of ED LOS reductions. Initially, there were real reductions in median ED LOS (total and reported) by the end of 2010. Our data from interviews and surveys help to pinpoint the actions and initiatives taken in the early period of policy implementation (from July 2009 to late 2010). Correspondingly, we can infer that initiatives taken in the later implementation period (from 2011 onwards) are only associated with reductions in reported ED LOS, rather than total ED LOS.

A detailed breakdown of the timing of case study initiatives into two periods is included in Table 5 below. As our interviews were predominantly with managers and clinicians within hospitals, our data does not provide a full picture of the range of ‘input’ solutions adopted by case study sites that sought to manage ED demand. We do know from other research that many demand-management initiatives – including attempts to increase the utilisation of after-hours medical clinics - were taken by case study DHBs [54]. However, the data on ED utilisation growth contained in Table 1 suggests that these strategies had little or no effect on ED demand. Our data from H1 identified some input strategies such as establishing a rest-home liaison nurse, and setting up a rapid access medical clinic based at the hospital which local primary care doctors were encouraged to use. While the rate of increase in ED utilisation was certainly lower in H1 than for other cases, this may have been attributable to slower population growth. For this reason our data in Table 5 focuses on initiatives taken *within* case study hospitals. The four columns in Table 5 represent the major categories of actions taken by hospitals. In the following section we discuss these in turn.

**Table 5:** What Hospitals Did to Reduce ED Length of Stay

	New Resources (staff and beds)	Improving Patient Flow	Better Information and Communication	Leadership and Social Marketing
Hospital 1 (H1)				
Early Implementation July 2009-Dec 2010	*Prior to target* QI project (2008): Staffing added ED nursing, flow coordinator trial (2008). *Post target* added/implemented: Staffing: ED, hospital, SSU - Added medical staff SSU and hospital (2010). Beds: ED, hospital, SSU - Reconfigurations of SSU adding ED observation beds and of medical/surgical wards to add medical (2010). Added bed to acute surgical unit (2010).	*Prior to target* QI project implemented 2008: ED Processes - ED fast track, care pathways for certain conditions. *Post target* implemented: ED processes - 3-2-1 model, changes in triage processes (2010), rapid assessment and direct SSU referral model (2010), ED discharge planning (i.e. discharge model including allied health), orderlies in ED (2010). Hospital and SSU processes - Discharge planning (i.e. hospital ward discharge, discharge lounge), reconfiguration of bed and clinical specialty models in SSU and hospital wards, daily bed management meetings (2010).	ED patient flow and reporting - IT screens upgraded for LOS (2010).	*Prior to target*: ED leadership of QI project. *Post target*: CEO leadership - New CEO focused on ED target & “front of house” (2010).
Later Implementation Jan 2011-Dec 2012	Staffing: ED, hospital, SSU Added ED, SSU nursing and medical staff, implemented aged care/hospital liaison role (2011–12).	Hospital processes: Added acute medical clinic for GP referral (2011). Additional acute operating theatre for flow (2012).	ED patient flow and reporting - Visible real time data in ED, hospital and SSU for clinicians/managers, target breach reports (2011). Bed information - Hospital/ED bed information improvements (2011).	Leadership - Separate hospital groups encouraged to include ED target focus (2011). Whole of hospital target leadership group (2012).
Hospital 2 (H2)	New resources (staff and beds)	Improving Patient Flow	Better Information and Communication	Leadership and Social Marketing
Early Implementation July 2009-Dec 2010	*Prior to target* QI project (2008): Staffing: added ED nursing and flow manager, added medical staff for new observation unit (2008). Beds: New acute observation unit (2008). *Post target* added/implemented: Staffing: ED, hospital, SSU - Added nursing staff ED, hospital, SSU, afternoon medical coordinator role. Added medical staff added SSU/ED, and orderlies (2009–10). Beds: ED, hospital, SSU - Increase and reconfiguration hospital medical, surgical ward beds (2009) and specialty SSU beds (2010).	*Prior to target*: QI project whole of hospital project including 6 h ED target (2008). *Post target* implemented: ED processes - 3-2-1 model, changes in triage processes, care pathways for certain conditions, rapid assessment and direct SSU referral model, ED discharge planning (i.e. discharge model including allied health), orderlies in ED (2009–10). Hospital and SSU processes - Ward and specialty SSU reconfiguration to increase beds and SSU hours, discharge planning (i.e. hospital ward nurse facilitated discharge at weekends, surgery discharge lounge), bed management meetings, cohorting patients for improved bed availability, afternoon medical coordinator, staff scheduling, changes (2009–10).	*Prior to target*: ED patient flow (2008): IT LOS screens upgraded (2008) *Post target* implemented: ED patient flow and reporting - Visible real time data in ED, hospital, SSU for clinicians/managers, target breach reports (2009–10). Bed information - Hospital/ED bed information improvements (2009–10). Communication devices - Cell phones, voice activated devices for medical, nursing, orderly staff (2010).	Leadership - Whole of hospital target leadership, clinical/management champions, and governance groups. Culture - Ready for target, strong QI and “can do” culture. Social marketing - Social marketing strategy group implemented and activity about target within whole hospital.
Later Implementation Jan 2011-Dec 2012		Patient flow and staff management – New hospital operations new unit management (2011).		
Hospital 3 (H3)	New Resources (staff and beds)	Improving Patient Flow	Better Information and Communication	Leadership and Social Marketing
Early Implementation July 2009-Dec 2010	Staffing: ED, hospital - Added ED nursing, ED orderlies, and hospital medical staff (2009). ED flow nurse, bed management coordinator and data analyst role for ED target project (2010).	ED processes - ED flow nurse and orderlies in ED (2009). ED Patient flow process engineering project with ED 3–2-1 model, flow coordinator, bed management coordination in ED and hospital (2010).	ED patient flow and reporting - Visible real time LOS data in ED, hospital, SSU for clinicians/managers. Results posted on ED/ hospital noticeboards (2010). Bed information - Hospital/ED bed information improvements (2010).	Leadership - Initially ED target implementation led by ED managers & clinicians.
Later Implementation Jan 2011-Dec 2012	Staffing: ED, hospital, SSU - Added nursing, medical staff for ED, hospital SSU (2011). Beds: ED hospital, SSU - New ED with additional beds (2011). Reconfiguration and additional medical ward beds (2011). Additional beds in children’s and medical SSUs (2012).	ED patient flow process engineering project (cont.) (2011) ED processes - Changes in triage processes, flow coordination strategies. ED frequent attenders strategy and low acuity redirect. ED and hospital staff scheduling changes (2011). Hospital, SSU processes - Ward and specialty SSU reconfiguration to increase beds (2011), extended SSUs hours (2012). Rapid assessment and direct SSU referral model. New Integrated Operations Centre (2011).	Communication devices - Cell phones, voice activated devices for medical, nursing, orderly staff (2010–11).	Leadership - Whole of hospital leadership group introduced (2011) but faltered, revised leadership group (2012). Social marketing - Social marketing activity re target within whole hospital (2011).
Hospital 4 (H4)	New Resources (staff and beds)	Improving Patient Flow	Better Information and Communication	Leadership and Social Marketing
Early Implementation July 2009-Dec 2010	Staffing: ED, hospital, SSU – QI improvement quality coach (2009) ED target coordinator (2010). Added ED, SSU nursing, medical staff, orderlies (2010).	ED processes - 3-2-1 model, changes in triage processes, ED nurse triage assessment, discharge planning (i.e. discharge safety checklist, ED lab requests prioritized), ED and hospital staff scheduling changes, orderlies in ED (2010). Hospital processes – Discharge planning (i.e, hospital laboratory system reorganized, hospital ward discharge lab requests prioritized, discharge planning safety checklist), weekend hospital clinics implemented (2010).	ED patient flow and reporting - IT LOS screens upgraded (2009). Visible real time LOS data in ED, hospital, SSU for clinicians/managers, daily breach reports (2010).	Leadership - ED target lead and monthly meetings with ED management (2010).
Later Implementation Jan 2011-Dec 2012	Staffing: ED, hospital, SSU - Added nursing, medical staff for ED, hospital, SSU (2011) Beds: ED hospital, SSU - New ED with additional beds (2011). SSU ward redeveloped & increased in beds, mixed specialty model (2012)	ED processes - Patient flow project (2011) Rapid assessment and direct SSU referral model, discharge planning (i.e. discharge planning model including allied health), ED orderlies (2011). Hospital and SSU processes – Discharge planning (i.e. hospital ward discharge lounge development) (2011). SSU review for patients with uncertain needs (2012).		Leadership - New hospital operations group (2011). Social marketing - Social marketing activity about target within whole hospital (2011).

*Abbreviations used in Table: ED* Emergency Department, *LOS* Length of Stay, *QI* Quality Improvement, *SSU* Short-Stay Unit, *GP* General Practice

#### New resources to help meet the target

New staff resources, particularly medical and nursing staff for the ED or SSU are a key response to the target in all of the case study hospitals, but with notable variation in the timing of their introduction and the number of staff. In H2, a new acute observation unit (separate to ED) was introduced in 2008 to manage acute medical presentations. In H3 and H4, the introduction of new beds was the product of existing plans to develop or build an ED and these facilities opened in both sites during 2011. There were no new bed resources introduced in H1 in response to the target.

The addition of new beds largely shaped the timing and allocation of new staff resources. In H2, these were introduced prior to the target, in 2008, with further increases during 2010. The new staff and bed resources that were introduced in the early implementation period in H1, H3 and H4 were predominantly for roles such as orderlies, ED flow nurses, and clinical staff dedicated to co-ordinating and monitoring the target. For H1, the new medical resources for ED and the short-stay unit were introduced in 2011 and 2012. In H3 and H4, the bulk of new staff resources became available when their new EDs opened in 2011.

All case study hospitals introduced new staff resources to the wider hospital, but the extent of these resources was much smaller compared to ED staff and beds. These new resources entailed a scattering of new nursing, hospital management, orderly and allied health resource to improve the flow of patients through the hospitals, as well as some new roles for Senior Medical Officers in wards. There was no clear pattern to the timing of additional resources for hospital wards.

#### Improving patient flow

Substantial strategic efforts were identified at all case study sites in order to move acute patients faster through the ED and hospital. This activity was based on a range of quality improvement programmes including clinical and operational process improvements. Most of these initiatives occurred in 2009 and 2010, the early implementation period. The reconfiguration of bed and clinical specialty models in short-stay units and hospital also supported this change. Within EDs, the use of the 3–2-1 model (where the patient journey for admitted patients is split into a 3-h ED assessment phase, a 2-h inpatient assessment phase and a 1-h transfer to ward phase) in H1, H3 and H4 promoted more timely management of admitted patients in the ED.

In H1, H2 and H3, improved rapid assessment practice and triaging of patients to ED and inpatient short-stay units enabled early movement of patients into parts of the hospital where the ED target did not apply. Process improvements were enhanced by new or changed clinical roles, for example, a new nursing coordinator for hospital medical wards on afternoon shifts in H2, a new target coordinator (nursing) role in the ED in H4, a new nursing coordinator role for patient flows in ED in H3 and a new ED Senior Medical Officer (SMO) role focused on patient flows in H4.

Improvements in hospital operations such as new hospital operations centres in H2 (2008, pre-target) and H3 (2011) and new roles such as aged care liaison in H1 (2011), were also aimed at moving patients through and out of the hospital in a timelier fashion. Reconfiguring medical rosters to better match and respond to the demand for acute patient care was also noted in all sites for hospital and ED services. However, there were some limits to the reach of patient flow initiatives. For example, ED clinicians in H3 struggled to get inpatient clinicians, particularly senior medical officers, to the table to develop a ‘whole-of-hospital’ response to the target. According to one ED clinician:

*So there is this group, and that, this group is trying to drive it and the trouble is, it’s attended predominantly by the converted and not the sinners. We’d love the sinners to come along.*

This meant that initiatives to improve patient flow that involved changes to inpatient services were less likely to gain traction in this hospital.

Better management of discharges from the ED and wards was noted in all hospitals including nurse facilitated weekend discharge in H2, a new hospital discharge lounge in H4, improved allied health service for ED and hospital discharge in H1 and H3. In H2, nurse facilitated weekend discharges helped to ensure that patients were not reliant on a medical visit on the weekend to be discharged, but rather an agreed plan put in place ahead of time that ensured a quality and timely process. In H1 and H3, improved allied health services enabled more timely discharges from the ED and hospital. In H4 a new discharge lounge helped to move patients from ward to the lounge to await their discharge, freeing up ward beds.

Across all of our case study sites, most process improvement activity occurred in the early implementation period of 2009–2010. In H2 there was advanced quality improvement capability and activity evident prior to the target’s introduction, more so than any of the other hospitals. In all sites, most of these changes took place within the ED and short-stay unit, although the changes to rostering and staffing provide evidence for process change across the hospital to achieve the target. Across most sites, changes to discharge processes and connections (handover) to aged care facilities were introduced to move patients more efficiently through the ED and hospital.

#### Information and ED LOS monitoring strategies

An important mechanism for achieving the target across all sites was the collection, analysis and utilisation of operational information regarding patient flows through the ED and hospital wards. The display of real time information on the target was also introduced at all hospital sites. This enabled clinicians and managers from many parts of the hospital to closely monitor delays to patient flow in the ED and the ability to anticipate target breaches. This monitoring was happening from surgeons and registrars in operating theatres in H4, from the Chief Executive Officer’s (CEO) desk in H1 and H4, and from group and service managers at H2 and H4. This activity and the information systems that supported it were implemented during 2010, and further initiatives based on this new capacity for real-time feedback were introduced in the later implementation period. Improvements in communication between staff and services were supported through the use of new technology such as cell phones for communicating admission information at H2, and voice activated devices for medical, nursing and orderly staff in H3.

#### Leadership and social marketing

Leadership and influencing culture change were key factors that supported and challenged the implementation of the target at the sites. Early and strong executive and senior clinical leadership of the target was readily apparent in H2 with a focus on promoting patient centred values and on whole of hospital commitment to the target.

At other sites, the key leadership initiatives undertaken in the early implementation period were quality improvement exercises led by senior ED clinicians and managers. The CEO of the District Health Board for H1 was strongly focused on the target, but conflict between him and ED clinical leaders became apparent in 2009 and 2010. In both H3 and H4 there was little evidence of senior DHB and hospital leadership of the target in the early implementation period. However, in 2011, H1, H3 and H4 each introduced whole of hospital leadership groups for the target.

Social marketing of the target was introduced in H2 in the early implementation period. For example whole of hospital forums, poster displays and production of videos on the target were three key methods of social marketing at this site. Staff at H3 and H4, described learning and developing their social marketing efforts from H2, with these efforts initiated in the later implementation period.

## Discussion

Our results show that the case study hospitals used a common range of strategies to implement the ED target. The mix, breadth and effects of these strategies varied across these sites. We suggest that the initial reduction in ED LOS (both reported and total) from 2009 to 2010 is mainly attributable to initiatives and additional resources introduced to improve patient flow, as these were implemented relatively early.

On the other hand, the introduction of additional beds and medical and nursing staff in EDs, new information and communication systems, and leadership strategies became more prominent later in the implementation processes for three of our case study sites, with only H2 adopting these approaches earlier. These initiatives can be linked to continued (slower) reductions in reported ED LOS, without having an impact on total ED LOS.

Our interview and survey data provide more detail to support this interpretation. A significant portion of the new staff and bed resources were used to build and support new short-stay units associated with newly refurbished EDs in H2, H3 and H4, and new protocols were developed to enable rapid assessment and direct SSU referral in all case study sites. New information and communication tools allowed ED staff to engage in real time monitoring to avoid target breaches. Being able to shift some patients to ‘off-target’ short-stay units became an important part of frontline staff responses to target pressure. From our interview data, this was evident in all hospitals, but was particularly pronounced in H3 and H4 where new beds were specifically created for this purpose. According to one clinician working in the ED in H4:

*I had to have some fights with people about people about not moving patients who weren’t clinically ready to go to the ward, and with the clinical director of the alpha ward (SSU) so there was some gaming going on…at times the pressure was, just move the patient to the alpha ward.*

This adds up to a plausible explanation for why the later implementation strategies had little impact on total ED LOS. Another possible interpretation of the absence of continued improvement in median total ED LOS is that there is a natural ‘floor’ level of about 3.5 h for this indicator. Although many strategies were consistent across all case sites, our qualitative data does suggest that some possible patient flow improvement strategies – particularly those that would involve changes to the admission practices of inpatient specialists – were not attempted by some hospitals. Thus, if there are limits to possible reductions in ED LOS, these may be due to the structural position and power of inpatient specialties. If this is the case, further improvement is considerably more difficult than successfully implementing process improvements involving equipment, nursing staff and orderlies.

This increasing reliance on short-stay units also occurred in the context of significant year-on-year increases in ED presentations in three of the four case study hospitals (H2, H3, H4). Clearly, SSUs made a discernible contribution to the management of patient flows within hospitals [46]. However, it is also clear that the creation and/or expansion of SSUs provided hospitals with the means of meeting the ED target, particularly once further opportunities for process improvement became scarce.

Our interview data provided evidence that transfers to short-stay units were *sometimes* without clinical justification, and that frontline ED staff did so particularly when there was significant organisational pressure to avoid target breaches, and decanting to short-stay units was the only option available. This in itself does not mean, though, that short-stays were exclusively or predominantly used for the purpose of avoiding target breaches. Short-stay units, be they located inside EDs, or elsewhere in the hospital, have evolved as an organisational response to increasing complexity. Even without a strong clinical justification for transfer to a short-stay unit, placing patients in these units is preferable to having them wait in ED corridors.

However, the increasing use of short-stay units has other implications for understanding hospital performance. While not all short-stay visits are captured in our data, the data that we do have suggests that successfully meeting the ED target may become increasingly irrelevant to the broader challenge of meeting increasing demand for acute and urgent care in hospitals. Given the increasing reliance some hospitals have placed on SSUs in the context of rising ED presentations, it is possible in the not-too-distant future that these units will reach capacity, and that the crowding problems of the mid 2000s will return. In this context, the future relevance of a throughput target solely focused on ED is questionable, and provides a possible example of what Christopher Pollitt [41] refers to as the ‘wearing out’ of a performance measure.

To sum up, our research suggests that initiatives to improve patient flow appear to have the most potential to create sustained improvements to ED LOS, particularly in conjunction with leadership strategies and increased resources. However, there appear to be limits to the extent of patient flow improvements that are possible. Strategies and practices adopted in the later implementation period based on improved information, feedback and monitoring are successful in reducing reported ED length-of-stay, but have no effect on total ED length-of-stay once short stay visits are taken into account.

## Study strengths and limitations

A key strength of this research is the mixed-methods approach that not only identifies trends using quantitative data, but builds coherent explanations of these trends based on qualitative data. As an observational study, we cannot definitively attribute changes in ED LOS to the introduction of the ED target as it is impossible to control for other influences and developments taking place simultaneously. However, any other major factor influencing the changes in ED LOS observed during this study period was considered unlikely. Another important limitation is that some interviewees and survey respondents could not precisely recall the timing of initiatives.

## Conclusion

Our analysis shows the value of a mixed-methods approach of integrating a range of data sources in order to provide a much fuller account of the implementation of the ED target in New Zealand. By incorporating qualitative data from interviews and surveys, we have been able to develop a plausible account of why ED length of stay reduced when it did.

Our main finding is that the New Zealand ED target was successful in reducing ED LOS, but there are some important caveats. Firstly, it appears that the influence of the target on the median ED LOS was confined to the period from mid-2009 to late 2010. Subsequent improvements in target performance are associated with reductions in ED LOS only if short-stay utilisation is excluded**.** This suggests that from 2011 onwards, improved target performance was achieved by increasing reliance on short-stay units as a tool for managing acute hospital demand. In this respect our findings reinforce those of Suzanne Mason and colleagues [2].

Our analysis lends support to the view that process improvements in ED can make a significant contribution to improved timeliness [55], and that the target was valuable inasmuch as it stimulated these improvements in patient flow. However, our analysis also questions the value of ED targets as a long term approach. To the extent that ED targets work in improving timeliness of care, we argue, it is in the form of a short, sharp shock. Given that ED demand continues to increase at rates above population growth rates [56] and increases in health system funding, additional policy and organisational strategies will be required in order to meet the challenges of increasing acute demand. Our research indicates the range and limits of what can be done within hospital settings. The next stage is to understand more fully the impact of strategies involving services beyond the hospital, and whether or not these help to reduce ED LOS.

Secondly, we have made plausible linkages between the initiatives taken by hospitals and health care organisations, and subsequent changes to median ED LOS. Our analysis shows that the introduction of the target in mid-2009 stimulated a range of initiatives, such as the 3–2-1 system, rapid assessment and triaging, and new clinical roles, to improve patient flow in the early implementation period. We suggest these initiatives were the ‘low-hanging fruit’ of ED target implementation in New Zealand. Thirdly, we showed that significant increases in ED bed and staffing capacity occurred in three of the four sites, and all sites made use of improved information and communication systems to improve flow through the hospital and generate real-time feedback on ED wait times. However, with the exception of one case study site (H2), the effects of these initiatives were not manifest until the later implementation period. These initiatives resulted in the increased use of short-stay units rather than reductions in total ED LOS. Finally, only H2 had a consistent and concerted strategy drawing on leadership and social marketing focusing on the target in the early implementation period. Concerted, strategic leadership developed later at the other three sites, *after* the reductions in total ED LOS had been achieved.

Taken together, these findings suggest that the target was influential in achieving reduced ED LOS in New Zealand. However, its value as a policy instrument depends to a considerable extent on the interpretation of an important means by which this was achieved. Specifically, the increased use of short-stay units was a crucial artefact of target implementation across all sites, and a key part of the strategies adopted in all case study hospitals, albeit to different extents. SSUs can be regarded as a more optimal use of hospital resources, but the use of the time target also means that sometimes SSUs will be used without clinical justification.

Taking a more global view, we also suggest that whatever value targets have as a mechanism to reduce waiting times in ED, this value is time-limited. While the target did stimulate reductions in the total median ED LOS in the first 12–18 months of implementation, these reductions plateaued thereafter. As such, the overall challenges in managing demand for acute and urgent care in New Zealand hospitals remain [31]. The growth in the utilisation of short-stay facilities also suggests that the target became less effective in ‘standing for’ the goal of improved timeliness of hospital care in the wake of increasing acute demand.

## Additional files

Additional file 1: Interviews Schedule for Qualitative Interviews. (PDF 246 kb)

Additional file 2: Survey of Initiatives Made and Resources Used to Help Meet the Shorter Stays in Emergency Departments Target. (PDF 516 kb)

## Abbreviations

25th: 25th percentile
3–2-1 model: Patient journey for admitted patients is split into 3-h ED assessment, 2-h inpatient assessment, 1-h transfer to ward
75th: 75th percentile
A&E: Accident & Emergency
CEO: Chief Executive Officer
DHBs: District Health Boards
ED LOS: Emergency Department Length of Stay
ED: Emergency Department
H2: Hospital 2
H3: Hospital 3
H4: Hospital 4
HI: Hospital 1
Hrs: hours
IQR: Interquartile range
LOS: Length of stay
Med: Median
NEAT: National Emergency Access Target
NHI: National Health Index
NZ: New Zealand
NZHIS: New Zealand Health Information Service
SMO: Senior Medical Officer
SSU: Short-Stay Units
