# Supplementary material for: New Zealand’s emergency department target – did it reduce ED length of stay, and if so, how and when?
Source: BMC Health Serv Res. 2017 Sep 26;17:678. doi: 10.1186/s12913-017-2617-1 (PMC5615466; doi:10.1186/s12913-017-2617-1)
Supplement: Supplementary file 1 — Interviews Schedule for Qualitative Interviews. (PDF 246 kb) [file 12913_2017_2617_MOESM1_ESM.pdf]

Interview Schedule for Managers

Adult Emergency Department
Auckland City Hospital

Health Systems Section

INTERVIEW SCHEDULE
(MANAGER)

NATIONAL PROJECT TITLE: Implementing performance improvement in New Zealand Emergency Departments: the six hour time target policy

NAME OF RESEARCHER STREAM 3: Linda M Chalmers

The aim of this research stream is to understand how the ED time target was and is being implemented across New Zealand's EDs and hospitals. To achieve this aim, front line health staff will be interviewed to identify perspectives, experiences and actions in relation to the target. Participants will be interviewed utilizing a semi-structured method based on the following questions:

1. What do you think about the target?
   - From your personal perspective
   - From your professional perspective
   - From your organisation's perspective

2. Based on your experience prior to the target's introduction, what difference, if any, has the target made to your management practice?

3. Based on your experience prior to the target's introduction, what difference, if any, has the target made to the service(s) you manage?

4. How have you enabled your service(s) to achieve the target?

5. How has/have your service(s) enabled achievement of the target?

APPROVED BY MULTI- REGION ETHICS COMMITTEE 04/09/10 FOR 3 YEARS - ETHICS REFERENCE NUMBER MEC 10/06/060.

Adult Emergency Department
Auckland City Hospital.

Health Systems Section

INTERVIEW SCHEDULE
(CLINICIAN)

NATIONAL PROJECT TITLE: Implementing performance improvement in New Zealand Emergency Departments: the six hour time target policy

NAME OF RESEARCHER STREAM 3: Linda M Chalmers

The aim of this research stream is to understand how the ED time target was and is being implemented across New Zealand's EDs and hospitals. To achieve this aim, front line health staff will be interviewed to identify perspectives, experiences and actions in relation to the target. Participants will be interviewed utilizing a semi-structured method based on the following questions:

1. What do you think about the target?
    - From your personal perspective
    - From your professional perspective
    - From your organisation's perspective

2. Based on your experience prior to the target's introduction, what difference, if any, has the target made to your clinical practice?

3. Based on your experience prior to the target's introduction, what difference, if any, has the target made to the service(s) you practice in?

4. How have you enabled your service(s) to achieve the target?

5. How has/have your service(s) enabled achievement of the target?

APPROVED BY MULTI- REGION ETHICS COMMITTEE 04/09/10 FOR 3 YEARS - ETHICS REFERENCE NUMBER MEC 10/06/060.
