# Supplementary material for: New Zealand’s emergency department target – did it reduce ED length of stay, and if so, how and when?
Source: BMC Health Serv Res. 2017 Sep 26;17:678. doi: 10.1186/s12913-017-2617-1 (PMC5615466; doi:10.1186/s12913-017-2617-1)
Supplement: Supplementary file 2 — Survey of Initiatives Made and Resources Used to Help Meet the Shorter Stays in Emergency Departments Target. (PDF 516 kb) [file 12913_2017_2617_MOESM2_ESM.pdf]

THE UNIVERSITY OF AUCKLAND
FACULTY OF MEDICAL AND HEALTH SCIENCES
School of Population Health

AUCKLAND DISTRICT HEALTH BOARD
Te Toka Tumai

Shorter Stays in Emergency Departments National Research Project

SURVEY

(Emergency Department Clinical Directors and Service Managers, DHB Management)

NAME OF RESEARCHERS: Dr Peter Jones, Dr Peter Carswell, A Prof Toni Ashton, Ms Linda Chalmers, Dr Susan Wells, Dr Tim Tenbensel, A Prof. Papaarangi Reid, Dr Elana Curtis, Ms Joanna Stewart, Prof Shanthi Ameratunga.

Thank you for helping with this research, which aims to investigate how the 'Shorter Stays in Emergency Departments' policy was implemented across New Zealand. The survey is in 4 sections. Section A covers changes made in ED, section B covers changes within the rest of the hospital and section C covers changes made in the wider community. Section D relates to DHB funding changes.

Please complete all sections that you believe apply to your role, or that you have knowledge of. If the section does not apply to you, please indicate the person in your DHB that you think would best be able to answer that section in the space provided. If there is insufficient space for you to complete your answer, please continue on a separate sheet.

Shorter Stays in ED Survey

1. **Site and Participant Information**

   1.1. DHB________________________________

   1.2. Site________________________________

   1.3. Role: ED Clinical Director ☐ ED Service Manager ☐
   Other ☐ State____________________________

The Following Questions Relate to The Introduction of the Shorter Stays in ED (SSED) Health Target in NZ in July 2009. Please tick the box that applies or enter free text where appropriate

2. **Who were the SSED Target Champion(s) in your Organisation? (Tick all that apply)**

No Champion ☐ CEO ☐ ED Clinical Director ☐ ED Service Manager ☐

ED Nurse Manager ☐ ED SMO ☐ Other Clinical Director ☐ Other Manager ☐

Other Nurse Manager ☐ Other SMO ☐

State which department if not ED____________________________

3. **When did your DHB start implementing changes to meet the SSED Target?____________________**

4. **When implementing the SSED target did your organisation consider how equity objectives with respect to Māori or other high need groups could be met?** **Yes** ☐ **No** ☐

A. CHANGES in the EMERGENCY DEPARTMENT

A.1. Did the Physical Structure of your ED Change in order to help meet the SSED Target?

Yes ☐ No ☐ Go to A.4

A.1.1. If Yes, please how did the physical structure of your ED change?

Physical Change	Tick if Applies	Number of Beds	Estimated Cost ($NZ)	Date Change Occurred (Month / Year)
A new ED was built		Old ED<br>New ED		
New bed spaces were made available to the existing ED				
A Short Stay ward was built in ED				
Existing ED beds were redesignated Short Stay				
Existing Other beds were redesignated Short Stay				

A.2. Did the physical structure change in ED for any other reason than to meet the SSED Target?

Yes ☐ No ☐ Go to A.3

A.2.1. If Yes, please indicate why the physical structure of your ED changed

____

A.2.2. If Yes, please indicate how the physical structure of your ED changed

____

A.2.3. If Yes, how many new beds were created? ____________________

A.3. Please Comment on Any other Physical Changes made to the ED help meet the SSED Target

THE UNIVERSITY OF AUCKLAND
FACULTY OF MEDICAL AND HEALTH SCIENCES
School of Population Health

AUCKLAND DISTRICT HEALTH BOARD
Te Toka Tumai

A.4. Were more Staff employed in ED to help meet the SSED Target?

Yes ☐ Please fill in the following table No ☐ Go to A.5

**Role**	**Full Time Equivalent (FTE)**	**Date Employed**<br>**(month/year)**
Senior Doctors		
Medical Officers Special Scale		
Registrars		
House Officers		
Clinical Nurse Specialists		
Emergency Nurse Practitioners		
Physicians Assistants		
Senior Nurses		
Nurses		
Health Care Assistants (Non Clinical)		
Orderlies		
Clerical Staff		
Managers		
External Consultants		
Māori Kaiawhina/Navigators		
Pacific social work/support		
Other (state)		

A.5. Were new roles created for ED staff to help meet the SSED Target?

Yes ☐ Please fill in the following table No ☐ Go to A.6

**Title**	**Role**	**FTE**	**Previous Role (this person)**	**Previous FTE (this person)**	**Date of Change (month/year)**
Bed Coordinator					
Flow Manager					
Breach Avoidance Facilitator					
Other (State Title)					
Other (State Title)					

A.6. Were staff changes in ED made for any other reason than to meet the SSED Target?

Yes ☐ No ☐ Go to A.7

A.6.1. If Yes, please indicate why the staff numbers/roles in your ED changed

A.6.2. If Yes, please indicate how the staff numbers/roles in your ED changed

A.7. If you have any other comment on ED Staff Changes that occurred around the time of the SSED target introduction please make them here.

THE UNIVERSITY OF AUCKLAND
FACULTY OF MEDICAL AND HEALTH SCIENCES
School of Population Health

AUCKLAND DISTRICT HEALTH BOARD
Te Toka Tumai

A.8. Process Changes in the ED

Please indicate the process changes that occurred in ED in order to help meet the SSED Target

**ED Processes**	**Tick if this process changed in your ED**	**FTE Used on this change**	**Estimated Cost ($NZ)**	**Date of Change** (month/year)
Triage				
Primary Nurse Assessment				
Nurse Treatment / Protocol				
Doctor Assessment / Protocol				
Allied Health Assessment / Protocol				
Availability of Investigations				
Admission Process				
Discharge Process				
Māori Specific Protocol / Process				
Pacific Specific Protocol / Process				
Other (State)				

A.9. Please describe how the ED processes changed and/or make any comments on process changes in the ED that occurred in order to help meet the SSED target.

A.10. If you feel someone else in your DHB is best equipped to answer Section A please state their name / contact details here:

B. CHANGES in the HOSPITAL

B.1. Did the Physical Structure of your Hospital change in order to help meet the SSED Target?

Yes ☐ No ☐ Go to B.2

B.1.1. If Yes, how did the physical structure of your hospital change? (tick all that apply)

**Physical Change**	**Tick if Applies**	**Type of Bed** (Medical, Surgical, Rehabilitation etc)	**Number of Beds**	**Estimated Cost ($NZ)**	**Date Change Occurred** (Month / Year)
More Short Stay bed spaces were built					
More Long Stay bed spaces were built					
More Rehabilitation bed spaces were built					
Other Physical Change (State)					

B.2. Did the physical structure change in your hospital for any other reason than to meet the SSED Target?

Yes ☐ No ☐ Go to B.3

B.2.1. If Yes, please indicate why the physical structure of your hospital changed

____

B.2.2. If Yes, please indicate how the physical structure of your hospital changed

____

B.3. Please Comment on Any other Physical Changes made to the Hospital help meet the SSED Target

B.4. Were more Staff employed in the Hospital to help meet the SSED Target?

Yes ☐ Please fill in the following table No ☐ Go to B.5

**Role**	**Full Time Equivalent (FTE)**	**Department(s)**	**Date Started (month/year)**
Senior Doctors			
Medical Officers Special Scale			
Registrars			
House Officers			
Clinical Nurse Specialists			
Emergency Nurse Practitioners			
Physicians Assistants			
Senior Nurses			
Nurses			
Health Care Assistants (Non Clinical)			
Orderlies			
Clerical Staff			
Managers			
External Consultants			
Māori Kaiawhina/Navigators			
Pacific social work/support			
Other (state)			

B.5. Were new roles created for Hospital staff to help meet the SSED Target?

Yes ☐ Please fill in the following table No ☐ Go to B.6

**Title**	**Role**	**FTE**	**Date Created** (month/year)	**Previous Role** (this person)	**Previous FTE** (this person)
Bed Coordinator					
Flow Manager					
Breach Avoidance Facilitator					
Other (State Title)					
Other (State Title)					
Other (State Title)					

B.6. Were staff changes in Hospital made for any other reason than to meet the SSED Target?

Yes ☐ No ☐ Go to B.7

B.6.1. If Yes, please indicate why the staff numbers/roles in your Hospital changed

B.6.2. If Yes, please indicate how the staff numbers/roles in your Hospital changed

B.7. If you have any other comment on Hospital Staff Changes that occurred around the time of the SSED target introduction please make them here.

B.8. Process Changes in the Hospital

Please indicate the process changes that occurred in the hospital in order to help meet the SSED Target

**Hospital Processes**	**Tick if this process changed in your Hospital**	**FTE Used on this change**	**Estimated Cost ($NZ)**	**Date of Change** (month/year)
Admission Process				
Primary Nurse Assessment				
Nurse Treatment / Protocol				
Doctor Assessment / Protocol				
Allied Health Assessment / Protocol				
Availability of Investigations				
Discharge Process				
Māori Specific Protocol / Process				
Pacific Specific Protocol / Process				
Other (state)				

B.9. Please describe how the processes changed in your hospital and/or make any comments on process changes in the hospital that occurred in order to help meet the SSED target.

B.10. If you feel someone else in your DHB is best equipped to answer Section B please state their name / contact details here:

C. CHANGES in the COMMUNITY

C.1. Did the Physical Structure of Community Healthcare change in order to help meet the SSED Target?

Yes ☐ Please fill in table No ☐ Go to C.2 Unknown ☐

C.1.1. If Yes, how did the physical structure of community healthcare change?

Physical Change	Tick if Applies	Type of Beds / Intervention / Service	Number of Beds / Services	Estimated Cost ($NZ)	Date Change Occurred (Month / Year)
More Rest Home Long Stay Residential Care bed spaces were built / opened					
More Accident and Medical Clinics were built / opened					
More General Practices were built / opened					
Interventions to prevent ED attendances were made					
Availability of Investigations					
Other Physical Change (State)					

C.2. Did the physical structure of community healthcare change for any other reason than to meet the SSED Target?

Yes ☐ No ☐ Go to C.3 Unknown ☐

C.2.1. If Yes, please indicate why the physical structure of community healthcare changed

____

C.2.2. If Yes, please indicate how the physical structure of community healthcare changed

____

C.3. Please comment on any other Physical Changes made to community healthcare to help meet the SSED Target

THE UNIVERSITY OF AUCKLAND
FACULTY OF MEDICAL AND HEALTH SCIENCES
School of Population Health

AUCKLAND DISTRICT HEALTH BOARD
Te Toka Tumai

C.4. Were more Staff employed in the Community to help meet the SSED Target?

Yes ☐ Please fill in table   No ☐ Go to C.5   Unknown ☐

**Role**	**Full Time Equivalent (FTE)**	**Site / Role**	**Date Started (month/year)**
General Practitioners			
Medical Officers Special Scale			
Geriatricians			
Clinical Nurse Specialists			
Emergency Nurse Practitioners			
Physicians Assistants			
Senior Nurses			
Nurses			
Health Care Assistants (Non Clinical)			
Clerical Staff			
Managers			
External Consultants			
Māori Kaiawhina/Navigators			
Pacific social work/support			
Other (state)			

C.5. Were new roles created for Community staff to help meet the SSED Target?

Yes ☐ Please fill in the following table No ☐ Go to C.6

**Title**	**Role**	**FTE**	**Date Created** (month/year)	**Previous Role** (this person)	**Previous FTE** (this person)

C.6. Were staff changes in the Community made for any other reason than to meet the SSED Target?

Yes ☐ No ☐ Go to C.7 Unknown ☐

C.6.1. If Yes, please indicate why the staff numbers/roles in your Community changed

C.6.2. If Yes, please indicate how the staff numbers/roles in your Community changed

C.7. If you have any other comment on Community Staff Changes that occurred around the time of the SSED target introduction please make them here.

C.8. Process Changes in the Community

Please indicate the process changes that occurred in the Community in order to help meet the SSED Target

**Community Processes**	**Tick if this process changed in your Community**	**FTE Used on this change**	**Estimated Cost ($NZ)**	**Date of Change** (month/year)
Admission Process				
Nurse Treatment / Protocol				
Doctor Assessment / Protocol				
Allied Health Assessment / Protocol				
Availability of Investigations				
Discharge Process				
Māori Specific Protocol / Process				
Pacific Specific Protocol / Process				
Other (state)				

C.9. Please describe how the processes in the Community changed and/or make any comments on process changes in the community that occurred in order to help meet the SSED target.

C.10. If you feel someone else in your DHB is best equipped to answer Section C please state their name / contact details here:

D. Funding Changes

D.1. Was your DHB budget allocation altered as a result of the SSED Target

Yes ☐ No ☐ Go to D.2 Unknown ☐

D.1.1. Please state how the budget allocation changed

Increased ☐ **Amount ($NZ)** ______________________

Decreased ☐ **Amount ($NZ)** ______________________

D.2. Did your DHB reallocate funding as a result of the SSED Target (2008-2012)?

Yes ☐ please fill in table No ☐ Go to D.3 Unknown ☐

Site of Care	Budget Increased<br>Yes/No	Amount Increased $NZ	Budget Decreased<br>Yes / No	Amount Decreased<br>$NZ
ED				
Hospital				
Primary Care				
Long Term Residential Care				
Māori Health				
Pacific Health				
Other				

D.3. Please make any other comments related to funding and budgets you think may be relevant to the SSED target

D.4. If you feel someone else in your DHB is best equipped to answer Section D please state their name / contact details here:
